# Reversible cerebral vasoconstriction syndrome following intracranial hypotension in a postpartum patient: a case report and literature review

**DOI:** 10.3389/fneur.2023.1281074

**Published:** 2023-10-12

**Authors:** Shuhua Li, Yi Yang, Jiacai Zuo, Ningli Du, Guoxian Kou

**Affiliations:** ^1^Department of Infectious Diseases, Mianyang Central Hospital, University of Electronic Science and Technology of China, Mianyang, China; ^2^Department of Neurology, Mianyang Central Hospital, University of Electronic Science and Technology of China, Mianyang, China; ^3^Department of Neurology, West China Hospital, Sichuan University, Chengdu, China

**Keywords:** intracranial hypotension, RCVS, cerebral vasospasm, postpartum, case report

## Abstract

**Introduction:**

Reversible cerebral vasoconstriction syndrome (RCVS) is a potentially life-threatening neurological disorder, rarely linked to intracranial hypotension. The presentation showed a patient with intracranial hypotension after peridural anesthesia who experienced RCVS during the early postpartum period, suggesting a potential involvement of intracranial hypotension in RCVS occurrence.

**Case report:**

A young female of 29 years of age initially developed an orthostatic headache after undergoing a painless delivery with lumbar epidural anesthesia. Intracranial hypotension was considered the underlying cause. Her headache was partially resolved after intravenous fluid therapy and strict bed rest. After 2 days, the patient had a new onset thunderclap headache with generalized seizures, cortical blindness, and elevated blood pressure. An MRI scan revealed high signal intensity within the temporal, parietal, and occipital lobes, left caudate nucleus, and right cerebellum on T2-FLAIR imaging with vasogenic edema. MR angiography indicated multifocal, segmental, diffuse narrowing affecting the cerebral arteries that are large and medium. An RCVS_2_ score was six, and the patient was diagnosed with RCVS. She was managed conservatively, quickly improving her symptoms. After 10 days, a follow-up MRI indicated a significant reduction in the abnormal signal, and a substantial resolution of the constriction of the cerebral artery constriction was confirmed by MR angiography.

**Conclusion:**

Intracranial hypotension could potentially lead to RCVS in postpartum patients, and it may be triggered by cerebral vasospasm secondary to intracranial hypotension.

## Introduction

Reversible cerebral vasoconstriction syndrome (RCVS) is a potentially fatal and uncommon disease (1). It is usually benign following a monophasic and self-limited process. However, some patients could suffer from catastrophic forms, including ischemic stroke, intracranial hemorrhage, cortical subarachnoid hemorrhage, posterior reversible encephalopathy syndrome (PRES), and death (2, 3). Small distal arteries are first affected by this vasospastic disease causing thunderclap headaches, hemorrhagic strokes, and PRES. Later, the disorder leads to ischemic strokes involving the medium and large arteries (4). RCVS has an unknown pathophysiological process, but endothelial dysfunction, sympathetic hyperstimulation, and alteration of vascular smooth muscle by oxidative stress have been postulated (5).

Intracranial hypotension is mainly presenting with an orthostatic headache accompanied by low opening pressure of cerebrospinal fluid (CSF) (<60 mmH20). Intracranial hypotension after the leakage of CSF may mechanically stimulate the arterial wall due to displacement of the brain anatomy. Moreover, the adrenergic system is activated, leading to cerebral vessel vasospasm and RCVS occurrence (6–8). Multiple potential etiologies of RCVS have been previously identified, but the association between intracranial hypotension and RCVS is rarely discussed. Limited reports have proposed RCVS may trigger by intracranial hypotension, and the pathophysiology is not clearly known at this time (6). Here, a postpartum patient was presented with intracranial hypotension after peridural anesthesia with subsequent RCVS.

## Case report

**Figure 1:**
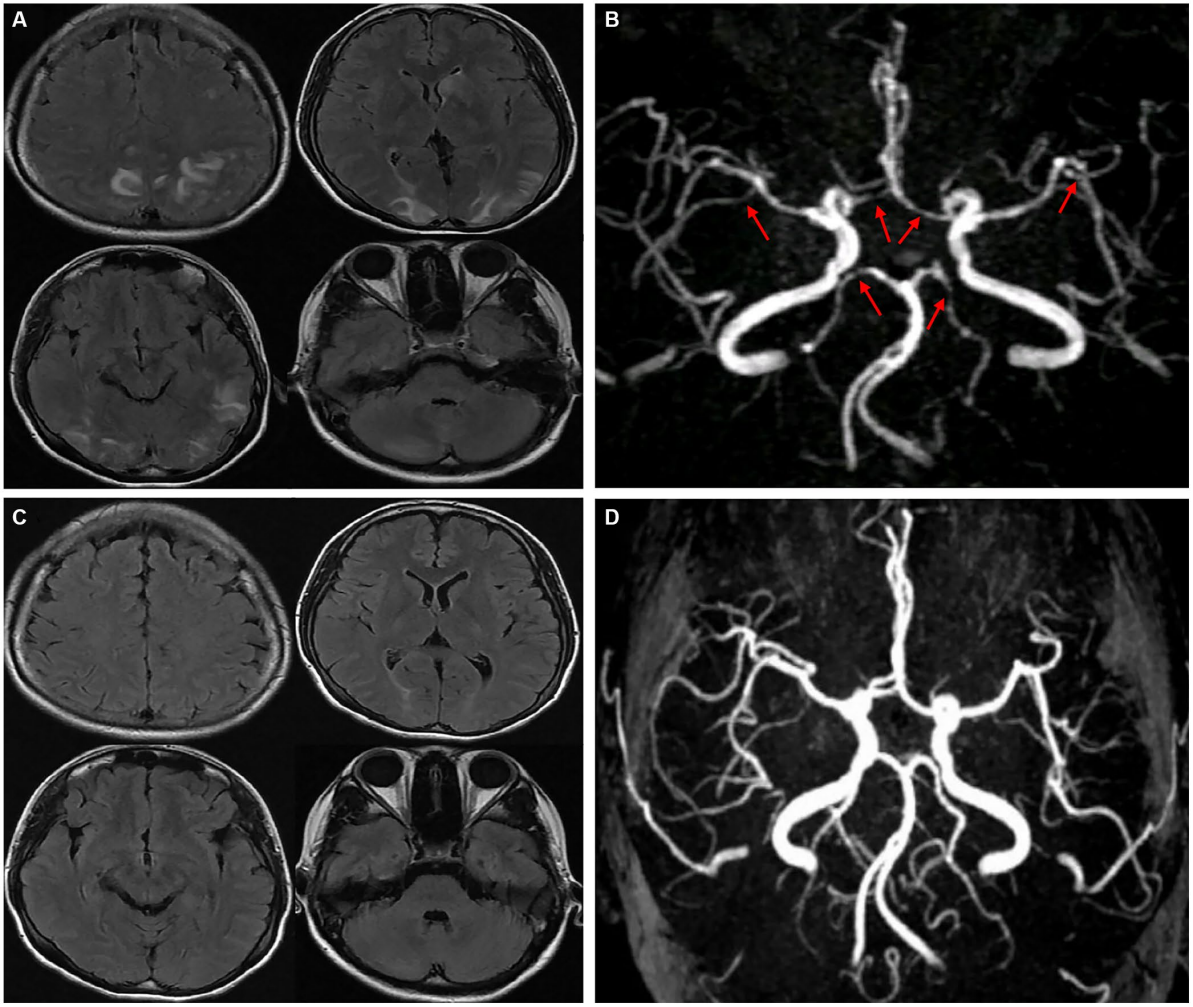
T2-FLAIR MRI revealed hyperintensities in the temporal, parietal, occipital lobes, left caudate nucleus, and right cerebellum **(A)**. An MRA indicated multifocal, segmental, and diffuse narrowing of cerebral arteries (large and medium) [**(B)**, red arrows]. After 10 days, complete remission of abnormalities was observed in a follow-up MRI compared to the previous signals **(C)**. An MRA demonstrated significant cerebral artery recovery from diffuse segmental constriction **(D)**.

**Figure 2:**
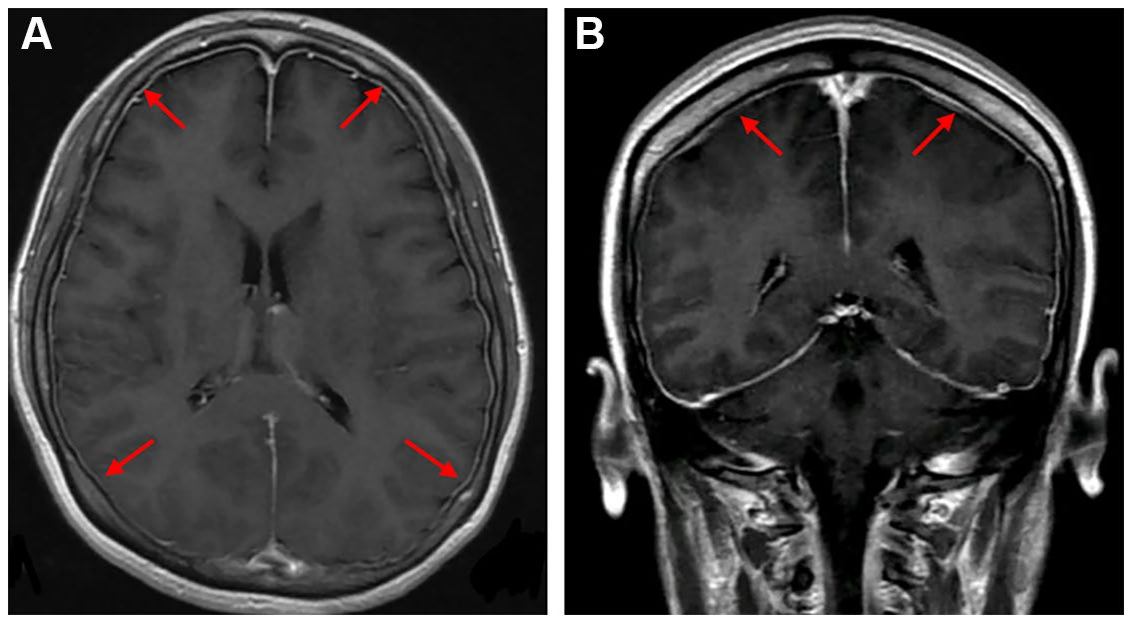
Axial and coronal gadolinium-enhanced T1-weighted MR images showed diffuse pachymeningeal enhancement [**(A,B)**, red arrows].

**Figure 3:**
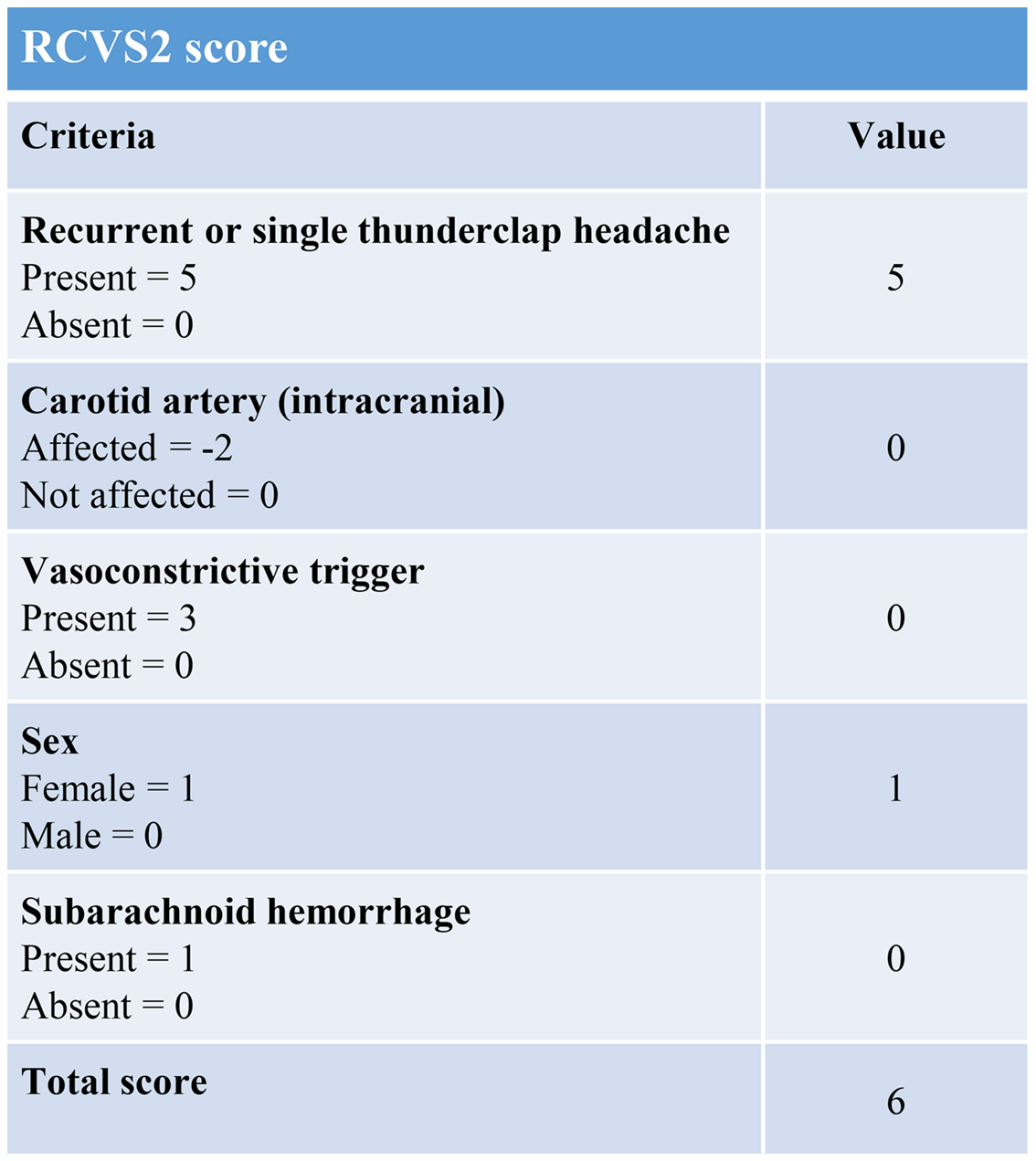
RCVS_2_ score for reversible cerebral vasoconstriction syndrome.

A young female who was 29 years old presented to the obstetrical department and underwent a painless delivery with lumbar epidural anesthesia at 39 weeks +6/7 gestation. After 8 hours of giving birth, the patient experienced an orthostatic headache. The cause was suspected of intracranial hypotension caused by CSF leakage due to an inadvertent epidural puncture. After receiving intravenous fluid therapy and strict bed rest, her symptoms were partially resolved in the next 2 days. However, the patient developed a new-onset thunderclap headache that peaked in less than a minute, lasting for more than 5 minutes, and differing from the previous headaches. She also reported blurred vision and recurrent generalized tonic–clonic seizures, requiring an emergency consult from a neurologist. She had vital signs with a blood pressure of 170/110 mmHg, a pulse rate of 92 beats/min, and a body temperature of 36.9°C. The neurological examination indicated cortical blindness, which was otherwise unremarkable. The complete blood counts, inflammatory markers, and standard chemistry panels were normal. The patient had a new-onset thunderclap headache with epileptic seizures and cortical blindness, suggesting a lesion in the cerebral cortex. An MRI scan revealed hyperintensities inside the temporal, parietal, and occipital lobes, left caudate nucleus, and right cerebellum on T2-FLAIR imaging (Figure 1A) without the restriction of diffusion displayed on diffusion-weighted imaging. Postpartum, the patient had an acute onset of symptoms with multiple brain lesions, indicating inflammatory or vascular disorders as the probable causes. Even though the intracranial infection could have been a possible cause, the patient had no fever, with normal inflammatory markers and negative meningeal signs. Additionally, the widespread enhancement of the dura mater indicated by the enhanced MRI could have been caused by low intracranial pressure syndrome (Figure 2), further decreasing the intracranial infection possibility. Cerebral vascular examinations were conducted to clarify the cause of the disease. Due to the peripartum status of the patient, cerebral venous sinus thrombosis was considered a possible cause. However, MR venography gave a normal result, leading to the exclusion of cerebral venous infarction. MR angiography indicated multifocal, diffuse, and segmental narrowing of arteries (both large and medium) within the anterior and posterior circulations, with occasional dilation of the segments (Figure 1B). The patient had an RCVS_2_ score of six (Figure 3). The clinical and imaging features were consistent with RCVS, and abnormal hypersignals on MRI FLAIR indicated vasogenic edema, depicting PRES. The patient was conservatively managed with continued bed rest and intravenous fluid infusion with a daily dosage of 2000 mL for 9 days. Moreover, a calcium channel antagonist (nifedipine 10 mg qd) and intravenous 10 mg diazepam followed by an antiepileptic drug (levetiracetam, 0.5 g bid) were also administered. These interventions quickly resolved her headache symptoms with vision recovery and no further seizures. After 10 days, another MRI was performed, which indicated markedly attenuated abnormal signals (Figure 1C). MRA showed that the cerebral arteries, which were diffuse and segmentally constricted, were improved significantly (Figure 1D).

## Discussion

RCVS is a neurovascular condition with characteristics of thunderclap headache which may or may not be associated with further neurological deficits that are focally placed. Additionally, RCVS is accompanied by seizures and diffuse segmental cerebral artery narrowing, improving within 3 months of onset (1). Approximately 70% of RCVS patients possess a known precipitating factor, with postpartum state, vasoactive drugs, and nasal decongestants being the primary recognized triggers (5, 9). A recent study demonstrated that the RCVS prevalence rate (11.9%) is relatively high among postpartum patients (10). However, a prospective study investigating 900 consecutive puerperae observed that the risk of puerperal RCVS is negligible, with only a 0.1% proportion (11). Therefore, intracranial hypotension may be crucial in RCVS pathogenesis other than the potential postpartum trigger.

Currently, the potential mechanisms concerning RCVS secondary to intracranial hypotension remain speculative. Chaves et al. (7) described a patient with diffuse cerebral vasospasm in intracranial hypotension and explained that severe CSF volume reduction could have triggered the vasospasm. Schievink et al. (12) presented a case with intracranial hypotension with severe, transient, and segmental cerebral arterial stenosis. They believed intracranial hypotension could be the underlying cause of reversible cerebral vasoconstriction. Caranzano et al. (13) indicated that intracranial hypotension could have been the primum movens of RCVS and cerebral venous sinus thrombosis. Cerebral vasospasm among patients with intracranial hypotension may be triggered by stimulating the arterial wall with mechanical force, which occurs by displacing the brain anatomy through CSF quantity reduction (7, 8). According to the doctrine provided by Monro-Kellie, the brain, CSF, and blood volume inside the cranium remain constant. CSF leakage leads to a decrease in intracranial volume. Thus, the intracranial venous system may be dilated as compensation, leading to adrenergic overstimulation and subsequent cerebral vessel vasospasm (6).

In a postpartum patient, positional headaches following epidural anesthesia and the presence of neurological signs guide the diagnosis of post-dural puncture headache (PDPH), occurring from intracranial hypotension due to leakage of CSF inside the epidural space through the location of dural puncture (14). PDPH may lead to neurological complications, such as persistent headache, subdural hematoma, depression, cerebral venous thrombosis, or bacterial meningitis (15). However, RCVS secondary to intracranial hypotension has limited reports. We suspected RCVS in our patient when the clinical headache features changed from positional to thunderclap headache (TCH) postpartum. RCVS is a common cause of TCH, except for subarachnoid hemorrhage (SAH), ischaemic stroke, cerebral venous sinus thrombosis, cervical artery dissection, intracranial infection and pituitary apoplexy (16). Moreover, RCVS can manifest as neurological symptoms, including seizures, altered mental status, disturbances in vision, ataxic gait, or motor and sensory insufficiencies (17, 18). Vascular RCVS imaging may indicate the alternating diffusing pattern of dilation and constriction of cerebral artery vessels while excluding other differentials (19). Additionally, 25–33% of RCVS patients may experience various complications, such as PRES, cortical subarachnoid hemorrhage, ischemic stroke, and intracranial hemorrhage (5, 20).

Advancement in imaging methods and clinical decision-making has made RCVS more recognizable. However, precisely diagnosing this disease could be challenging. The differential diagnosis of cerebral arteriopathies often includes RCVS and primary angiitis of the CNS (PACNS). PACNS is treated with immunosuppressive drugs for a prolonged time, but steroids in RCVS could have deleterious effects (21). Female gender, migraines, and postpartum are usual in RCVS patients. Additionally, TCH, infarcts at the border regions, cortical SAH, and vasogenic edema highly predict RCVS. However, PACNS patients may have several tiny and deep infarcts, white matter lesions that are quite deep, lesions resembling tumors, or various lesions elevated by gadolinium (9, 22). Furthermore, an RCVS_2_ score of ≥5 demonstrated high sensitivity and specificity for detecting RCVS, while a ≤ 2 effectively excluded it (23). Vasogenic edema resembling PRES can be identified across 9–38% of RCVS patients, consistent with our case (24). Over one-third of RCVS cases possess increased headache-induced blood pressure. Enhanced pressure of blood within the arteries and vasoconstrictions in the major cerebral vessels were essential PRES identifiers in such patients (25, 26).

No official guidelines exist for RCVS therapy. RCVS patients must avoid headache triggers, withdraw precipitating vasoactive agents, and require calcium channel antagonists to reduce TCH (25). In our case, intracranial hypotension may be the primum movens of RCVS. Most patients with intracranial hypotension respond to a short conservative management course followed by non-targeted patching of epidural blood (27). RCVS drug treatment with antagonists of calcium ion channels (nifedipine, verapamil, or nimodipine) positively impacts the clinical progression. However, glucocorticoids can lead to adverse outcomes (24).

## Conclusion

In summary, new-onset headaches should be carefully assessed for secondary headaches during the peripartum period. This study presented a postpartum RCVS patient after intracranial hypotension due to a CSF leakage caused by an inadvertent epidural puncture. Thus, due to cerebral vasospasm, intracranial hypotension could trigger RCVS.

## Data availability statement

The raw data supporting the conclusions of this article will be made available by the authors, without undue reservation.

## Ethics statement

The studies involving humans were approved by Institutional Ethics Committee of Mianyang Central Hospital. The studies were conducted in accordance with the local legislation and institutional requirements. The patients/participants provided their written informed consent to participate in this study. Written informed consent was obtained from the participant/patient(s) for the publication of this case report.

## Author contributions

SL: Writing – original draft, Writing – review & editing, Data curation, Software. YY: Writing – original draft, Writing – review & editing, Conceptualization. JZ: Investigation, Methodology, Software, Writing – review & editing. ND: Formal analysis, Project administration, Writing – review & editing. GK: Conceptualization, Resources, Supervision, Validation, Visualization, Writing – original draft.
